# An observational study of the impact of COVID-19 and the rapid implementation of telehealth on community mental health center providers

**DOI:** 10.1186/s43058-021-00123-y

**Published:** 2021-03-11

**Authors:** Marisa Sklar, Kendal Reeder, Kristine Carandang, Mark G. Ehrhart, Gregory A. Aarons

**Affiliations:** 1 University of California San Diego Department of Psychiatry, 9500 Gilman Drive (0812), La Jolla, CA 92093-0812 USA; 2 Child and Adolescent Services Research Center, 3665 Kearny Villa Rd., Suite 200N, San Diego, CA 92123 USA; 3 UC San Diego Dissemination and Implementation Science Center, Altman Clinical and Translational Research Institute, San Diego, USA; 4 University of California Los Angeles Department of Psychology, Psychology Building 1285, Box 951563, Los Angeles, CA 90095-1563 USA; 5 University of Central Florida Department of Psychology, P.O. Box 161390, Orlando, FL 32816-1390 USA

**Keywords:** COVID-19, Telehealth, Community mental health, Provider perspectives, Evidence-based practice

## Abstract

**Background:**

The COVID-19 pandemic has remarkably altered community mental health service delivery through the rapid implementation of telehealth. This study reports provider perspectives on the impact that COVID-19 and the transition to telehealth had on their work and their ability to deliver evidence-based practices (EBPs).

**Methods:**

Providers (*n* = 93) completed online surveys with quantitative measures and open-ended items exploring their reactions to COVID-19 and to the transition to providing services via telehealth.

**Results:**

Perceptions of personal risk and rumination around COVID-19 were low, while telehealth was viewed positively by providers. Three major themes emerged regarding the major impacts of COVID-19 on work: (1) the altered nature of interactions between patient/client and provider due to telehealth implementation, (2) changes in provider expectations regarding productivity, and (3) challenges maintaining work-life balance. In regard to the major impacts of COVID-19 on EBP delivery, three themes emerged: (1) increased difficulty delivering certain therapies via telehealth, (2) potential limitations to session confidentiality, and (3) challenge of engaging children in telehealth.

**Conclusions:**

In the context of the COVID-19 pandemic, community mental health providers continued to engage with clients and deliver EBPs while navigating a number of changes related to the rapid transition to and implementation of telehealth. This study highlights the need for further work on what supports providers need to effectively engage with clients and deliver EBPs via telehealth, and has implications for how telehealth is sustained or de-implemented post-COVID-19.

**Supplementary Information:**

The online version contains supplementary material available at 10.1186/s43058-021-00123-y.

Contributions to the literature

- In response to 2019-nCoV/COVID-19, community mental health centers (CMHCs) rapidly implemented telehealth services to minimize the risk of COVID-19 transmission.
- This report introduces new and adapted measures to explore providers’ experiences in making this transition to telehealth and providing services in the context of a pandemic.
- As services continue to be offered via telehealth, CMHCs should consider strategies for supporting providers’ ongoing delivery of EBPs via telehealth to optimize treatment for clients.

## Background

The novel coronavirus disease (2019-nCoV/COVID-19) drastically impacted the context in which mental health services are provided. Many community mental health centers (CMHCs) rapidly implemented telehealth services in order to comply with ‘stay at home’ restrictions set across the country [1]. Mental health providers were subsequently tasked with transitioning their services, including the delivery of evidence-based practices (EBPs), from in-person treatment to telehealth. Although evidence for the effectiveness of telehealth services (i.e., services provided via phone and/or video platforms) has existed for over 20 years, numerous barriers have delayed its widespread use until now [2–4]. In response to COVID-19, a nationwide telehealth implementation effort occurred in only a matter of weeks. This rapid telehealth implementation [5] directly contrasts with the 17-year research-to-practice pipeline [6] and is even more rapid than the 3-year length of time EBPs are integrated into real-world settings with the aid of an implementation team [7]. In accordance with the STROBE checklist of items included in reports of observational studies (Additional file 1), this paper reports on the impact of the COVID-19 pandemic and rapid implementation of telehealth services from the perspectives of CMHC providers in one Midwest state and discusses implications regarding the quality and sustainment of rapid implementation efforts.

## Method

### Participants and setting

Data were collected as part of an ongoing service contract between the UC San Diego and the statewide behavioral health services system in one Midwest state to engage the state and their CMHCs in the Leadership and Organizational Change for Implementation strategy [8, 9]. Participants were behavioral health services providers (*n* = 93, response rate = 77%) from 6 CMHCs that were also contracted to improve upon provider delivery of combined motivational enhancement therapy and cognitive behavioral therapy (MET/CBT) and other EBPs. In response to COVID-19, CMHCs implemented telehealth services, typically including both video conference and telephone modes of delivery. Providers identified mostly as female (*n* = 77; 84.6%), non-Hispanic (*n* = 81; 89%), and White (*n* = 79; 86.8%) and were 41 years old on average (*sd* = 14.8 years). Most providers had completed master’s level education (*n* = 65; 71.4%) and identified social work as their primary discipline (*n* = 44; 48.4%). Providers reported spending the greatest percentage of their work time in psychotherapy and/or counseling ($\overline{x}$=44.1%) and reported an average caseload of 52.1 (*sd* = 39.5) clients per month. See Table 1 for provider demographics.

**Table 1:** Provider demographics

**Age (years;** $\overline{\mathbf{x}}$** ± sd)**	41.0 ± 14.8	
**Gender**	***n***	**%**
Female	77	82.8
Male	13	14.0
Other	1	1.1
Missing	2	2.2
**Race**	***n***	**%**
White	79	84.9
Black or African American	3	3.2
Asian	2	2.2
American Indian/Alaska Native	1	1.1
More than one race	6	6.5
Missing	2	2.2
**Ethnicity**	***n***	**%**
Non-Hispanic	81	87.1
Hispanic	10	10.8
Missing	2	2.2
**Highest level of education**	***n***	**%**
Some college	1	1.1
College graduate	14	15.1
Some graduate work	5	5.4
Master’s degree	65	69.9
PhD, MD, or equivalent	6	6.5
Missing	2	2.2
**Primary discipline**	***n***	**%**
Drug/alcohol counseling	13	14.0
Social work	44	47.3
Child development	2	2.2
Marriage and family therapy	2	2.2
Psychology	16	17.2
Other	14	15.1
Missing	2	2.2
**Providers per agency**	***n***	**%**
Agency 1	7	7.9
Agency 2	15	16.9
Agency 3	11	12.4
Agency 4	11	12.4
Agency 5	43	48.3
Agency 6	2	2.2
**Years at present agency (**$\overline{\mathbf{x}}$ **±** ***sd*)**	4.7 ± 7.7	
**Years in present position (**$\overline{\mathbf{x}}$ **±** ***sd*)**	3.2 ± 5.9	
**Percentage of your work time doing the following:**	$\overline{\mathbf{x}}$ **±** ***sd***	
Standardized assessments	7.1 ± 10.6	
Case management	12.0 ± 18.6	
Psychotherapy and/or counseling	44.1 ± 28.6	
Administrative work (e.g., documentation, billing)	18.1 ± 11.9	
Meeting with your supervisor	7.2 ± 10.6	
Supervising others	4.3 ± 13.4	
Travel	2.1 ± 5.0	
Other	5.1 ± 14.5	

### Procedures and measures

CMHC providers completed surveys via the Qualtrics web-based platform that included measures targeting their reactions to the COVID-19 outbreak and transition to providing services via telehealth. The measures that were utilized are described below. Unless otherwise noted, participants responded to each measure using a 5-point response scale ranging from 0 = “Strongly Disagree” to 4 = “Strongly Agree.” When appropriate, the internal consistency of each measure was assessed for this sample using Cronbach’s *α*; results are included in Tables 2 and 4.

**Table 2:** Survey scale descriptive statistics

	Cronbach’s *α*	Minimum	Maximum	Mean	Std. deviation	Test of between-agency differences
Perceptions of personal risk	.87	0.00	4.00	1.41	0.83	*F*(5, 83) = .89, *p* = .492
COVID-19 rumination	.81	0.00	3.33	1.10	0.87	*F*(5, 83) = 1.13, *p* = .352
Burnout	.91	0.00	4.00	2.31	0.97	*F*(5, 83) = 1.84, *p* = .114
Perceived organizational support	.93	0.00	4.00	2.66	1.00	*F*(5, 83) = .86, *p* = .509
Telehealth self-efficacy	.92	0.00	4.00	2.68	0.85	*F*(5, 83) = .99, *p* = .426
Collective efficacy	.76	0.67	4.00	2.55	0.77	*F*(5, 83) = .90, *p* = .483
Telehealth beliefs	.85	0.00	4.00	2.89	0.84	*F*(5, 83) = 2.22, *p* = .060

#### Perceptions of personal risk

This 9-item measure was adapted from Wu et al.’s measure assessing perceptions of personal risk around SARS [10]. Items were adapted to assess participants’ perceived risk of being exposed to, and getting infected with, COVID-19.

#### COVID-19 rumination

This 3-item measure was developed by LeNoble and colleagues to assess participants’ rumination about COVID-19’s interference with their work [11].

#### Work changes due to COVID-19

Based on feedback from providers and their leaders, three distinct types of work changes were identified. A single item was developed to capture each type of work change through an iterative process of item generation, discussion, and refinement until consensus on item wording was achieved. The resulting three items assessed changes in tasks, settings, and teams that mental health providers experienced following the COVID-19 outbreak. Participants responded using a 5-point response scale ranging from 0 = “Not at all” to 4 = “Very great extent.” Because the three items were developed to capture three distinct types of work changes and were not intended to represent a single underlying construct, internal consistency was not assessed [12].

#### Burnout

The Copenhagen burnout inventory assesses participants’ emotional exhaustion and work-related frustration [13]. Participants responded to three items targeting work-related burnout within the past 2 weeks using a 5-point scale ranging from 0 = “Never” to 4 = “Always.”

#### Perceived organizational support

We used three items from Eisenberger et al. to assess perceptions of the helpfulness, care, and concern of the agency for providers [14].

#### Telehealth self-efficacy

This 4-item measure adapted from a measure developed by Lau and Brookman-Frazee assessed participant’s confidence, knowledge, understanding, and preparation to deliver therapy via telehealth [15, 16].

#### Collective efficacy

This 3-item measure adapted from Jex and Bliese assessed efficacy beliefs targeting the agency’s transition to telehealth [17].

#### Telehealth beliefs

This 5-item measure adapted from the University of Michigan’s Behavioral health Workforce Research Center assessed whether providers had a positive view of telehealth [18].

#### Transition to telehealth

Seven items evaluating the transition to telehealth were developed by the study authors through an iterative process of item generation, discussion, and refinement until consensus on item wording was achieved. These items measured the extent to which different aspects of treatment were better or worse when serving clients via telehealth as opposed to in-person treatment; see Table 4 for the individual items. Participants responded using a 5-point response scale ranging from 0 = “Significantly worse with telehealth relative to in-person” to 4 = “Significantly better with telehealth relative to in-person.”

#### Open-ended survey questions

Participants responded to two open-ended survey items. The first asked participants about the major impacts of COVID-19 on their work, and the second asked about the major impacts of COVID-19 on the use of a specific EBP (MET/CBT) and other EBPs in general.

### Analysis

Participant responses were aggregated across all items to obtain an overall scale mean with the exception of the work changes and the transition to telehealth items, which were analyzed individually. Descriptive statistics of quantitative measures were assessed to explore providers’ responses and/or reactions to the COVID-19 outbreak, and subsequent transition to providing services via telehealth. Potential between-agency differences in provider responses were explored using univariate analysis of variance (UNIANOVA). Provider responses to open-ended survey questions were analyzed using a template organizing style of interpretation. Specifically, responses were first reviewed by authors (MS, KR, and KC) to gain familiarity with the content and to isolate broad themes. Text was then sorted and organized in accordance with broad themes, and new themes were generated when appropriate. All authors held meetings to review “chunks” [19] of text and develop summaries of findings.

## Results

### Quantitative items

See Tables 2, 3, and 4 for survey item and scale descriptive statistics. On average, provider item and scale scores were lowest on COVID-19 rumination ($\overline{x}$ = 1.10) and perceptions of personal risk ($\overline{x}$ = 1.41). Provider item and scale scores were greatest on the telehealth beliefs scale ($\overline{x}$ = 2.89) indicating generally favorable beliefs and attitudes about telehealth, followed by telehealth self-efficacy ($\overline{x}$ = 2.68) and perceived organizational support ($\overline{x}$=2.66). With regard to the work changes (Table 2), the highest scores were for changes in the work setting ($\overline{x}$=3.66), followed by changes in the work tasks ($\overline{x}$=2.91). Fewer changes were reported for the work team ($\overline{x}$ = 2.11). With regard to the questions evaluating the effects of telehealth (Table 4), providers reported the largest benefits for scheduling ($\overline{x}$ = 3.12), and the biggest challenge with patient/client focus ($\overline{x}$ = 2.38). Results from UNIANOVA indicated no significant differences between agencies in any of the survey items and/or scale scores (see Table 2).

**Table 3:** Work changes due to COVID-19 descriptive statistics

	Minimum	Maximum	Mean	Std. deviation
Because of COVID-19, my work tasks have changed.	0	4	2.91	1.09
Because of COVID-19, my work setting has changed.	1	4	3.66	0.64
Because of COVID-19, my work team has changed.	0	4	2.11	1.45

**Table 4:** Transition to telehealth descriptive statistics

	Minimum	Maximum	Mean	Std. deviation
Relationships between you and your patients/clients	1	5	2.80	0.71
Quality of communication between you and your patients/clients	1	5	2.57	0.81
Rate of no-shows with fewer being better	1	5	2.81	1.22
Patient/client focus during sessions	1	5	2.38	0.85
Patient/client engagement in treatment	1	5	2.71	0.88
Confidentiality of discussions with patients/clients	1	5	2.82	0.78
Patient/client willingness to schedule sessions	1	5	3.12	1.00

Responses ranged from 0 = “Significantly worse with telehealth relative to in-person” to 5 = “Significantly better with telehealth relative to in-person.” Internal consistency of this measure was high at Cronbach’s *α* = .82

### Qualitative items

#### Major impacts of COVID-19 on work

Analysis of open-ended item responses provided by 89 unique participants identified three major themes (see Table 5). One centered on the impact that the transition to telehealth had on interactions with patients/clients. Providers described technological barriers to high quality interactions identifying challenges such as “blocked cell number,” “caseload lives in rural areas…not all kids have access to internet or stable internet,” and “some clients do not have technological capacity for video conferencing.” Some providers commented on the challenge of developing/maintaining rapport through the transition to telehealth. For example, providers reported “not being able to build rapport with new clients” and that “not being able to provide therapy in person and be able to read client’s body language has been the major impact.” Some providers reported beliefs that telehealth facilitated improvements in communication with clients. For example, providers stated that “it has been more enjoyable regarding relationships with clients…due to the less formal atmosphere as clients are more comfortable in their homes,” and that they experienced “stronger communication” with clients.

**Table 5:** Provider responses and themes regarding major impacts of COVID-19 on work

Theme		Provider responses
Patient/client and provider interactions	Technology	My caseload lives in a rural area so not all kids have access to internet or stable internet.
		Blocked cell number.
		No response.
		Lack technology.
	Developing and/or maintaining rapport	Not being able to build rapport with new clients—it’s difficult to introduce yourself via telephone or even video.
		It is difficult to have longer appointments.
		It has been difficult to fully engage families as I have been able to before.
		I used to have very high guardian engagement, and for me, this has decreased.
		It has been very difficult to see my clients consistently. A lot of the people I work with are students, and unfortunately, some of the parents don’t hold their children accountable for doing video sessions.
		My clients’ participation has gone down tremendously.
	Communication	It is difficult for students to pay attention and they are easily distracted when I am trying to have sessions with them.
		It is sometimes difficult to connect with the client or to judge their state of mind without the visual clues.
		Sessions are shorter.
		It has also been more enjoyable regarding relationships with clients. I think it may be due to the less formal atmosphere as clients are comfortable in their homes.
Productivity expectations	Reduced productivity	My productivity has dropped because I have been having issues coordinating with some families.
		Less productivity hours.
		Loss of productivity, increased phone calls and chasing clients, helping clients download Zoom, Skype, etc.
	Increased demands	Increase in documentation required.
		Increase pressure regarding productivity and revenue.
		More paperwork without receiving productivity for increased time spent.
Work-life balance	General work-life balance	Lack of home life/work life balance, stressful, time management is difficult.
		My work-life balance has been disrupted.
		Working much later in the evening.
	Working at home with children	Working from home while taking care of my child makes it difficult to do my best work with clients.
		It can be difficult to work from home with children.

Another theme centered on the changes in provider expectations regarding productivity. Some providers commented on reduced productivity due to this transition, stating “my productivity has dropped…because I have been having issues coordinating with some families” and “I used to have very high guardian engagement…this has decreased; guardians have limited to no access to technology at times.” Some providers commented on increased demands like “increase in documentation required,” “increased pressure regarding productivity and revenue,” and “more new changes in documentation and more paperwork without receiving productivity for increased time spent.”

A third theme centered on challenges maintaining a work-life balance. For example, providers stated that “work-life balance has been disrupted as I have difficulty separating myself from work” and “lack of home life/work balance…time management is difficult.” Providers also reported challenges with working from home while caring for their children, stating “working from home while taking care of my child makes it difficult to do my best work with clients” and “it can be difficult to work from home with children.”

#### Major impacts of COVID-19 on EBP delivery

When asked about the major impacts of COVID-19 on delivery of EBPs, three themes emerged based on responses from 86 unique participants (see Table 6). One centered on the challenge delivering certain therapies via telehealth wherein providers stated “barriers to implementing services like play therapy are somewhat dependent on the setting,” “prize draws and drug screens are difficult to do,” “lack of client ability to access the worksheets or use a video format,” and that they “haven’t tried [Eye Movement Desensitization and Reprocessing]-bilateral stimulation” via telehealth.

**Table 6:** Provider responses and themes regarding major impacts of COVID-19 on implementation of evidence-based practices (EBPs)

Theme		Provider responses
Modality specific challenges	Play therapy	Barriers to implementing services like play therapy that are somewhat dependent on the setting.
	Eye movement desensitization and reprocessing (EMDR)	Haven’t tried EMDR bilateral stimulation.
		Clients discomfort rating Outcome and Session Rating Scale (ORS/SRS) verbally.
		I cannot do EMDR treatment—clients not preferring to try the phone way of doing it.
	Contingency management / Combined MET/CBT	Prize draws and drug screens are difficult to do.
		Can’t supply “rewards.”
		Can’t administer urine drug screens to assess for substance use.
	Group therapy	My Intensive Outpatient treatment and Relapse Prevention groups don’t feel as productive using Zoom.
		Interaction with each other is more difficult.
		People that were in an Intensive Outpatient Program group are now contacted 1-2 times weekly by an LCSW that may not have addictions experience of the level of experience needed. So clients are missing out a lot on the value of group therapy.
		No longer facilitating a group and having to contact clients individually, which has been time consuming.
	Therapy materials / Worksheets	Lack of client ability to access the worksheets
		Not being able to hand the client a cognitive distortion list.
		Can’t share worksheets with patients
		Client is not able to fill out [assessment] themselves.
		Getting materials to patients is basically not happening due to the level of tech availability and ability.
		I don’t have access to the VPN from home so I have to save everything myself into a folder and remember to send to clients before/during/after appointments.
Confidentiality	Privacy	Many clients have less privacy at home—may have partners/kids around
	Trauma	Some client’s [diminished] willingness to process trauma.
		I have not been able to carry out TF-CBT properly due to not being able to meet with some of my patients via face-to-face even if it is virtual.
		I am uncomfortable with them on healing their trauma due to not being able to see if they are upset, being triggered, etc.
Children	Engaging children in telehealth	It is difficult to teach my students over the video sessions at times depending on the subject we are discussing and distractions.
		Conducting sessions via video or telephone has created some difficulties for young clients that struggle with inattention.
		Younger kids often engage better face to face.
	Children with complex behavioral health needs	It is difficult to explain it to an adolescent, especially ones with learning disabilities/lower IQ over the phone.
		Most of my clients are elementary school age children who need behavioral management services and substantial support. Doing this over the computer instead of face to face is not optimal.

A second theme centered on the potential limitations to confidentiality and/or lack of privacy when providing treatment with telehealth. Providers reported challenges engaging clients in EBP for the treatment of trauma because some clients are less willing to discuss traumas due to limited confidentiality/privacy. Some providers stated “many clients now have less privacy at home—may have partners/kids around;” other providers reported discomfort processing trauma without being able to see how their clients are responding stating that “I have not been able to work with some of my patients on healing their trauma…I am uncomfortable due to not being able to see if they are upset, being triggered, etc.”

The final theme that emerged centered on the general challenge of engaging children in treatment via telehealth. Providers stated “it is difficult to teach my students over the video sessions at times depending on the subject we are discussing and distractions,” “some parents don’t hold their children accountable for doing video sessions,” “parents prefer I work with their children face-to-face,” and that “younger kids often engage better face to face.”

## Discussion

This study investigated major impacts of COVID-19 and the rapid implementation of telehealth services on CMHC providers’ work and EBP delivery. Consistent with a recently proposed definition of rapid implementation, telehealth services were provided to those in need, with speed and efficiency, through a pragmatic reconceptualization of rigor [5]. CMHCs and representative providers utilized various modes of telehealth delivery including both video conference (e.g., via Zoom, Skype, Cisco Webex) and telephone. For clients unable to access videoconferencing sessions, providers typically reported using telephone. Providers viewed CMHC transitions to telehealth positively and reported confidence in their abilities to deliver services via telehealth. Notably, providers held generally favorable views of telehealth. Provider acceptance of telehealth services has been found to play a primary role in the implementation and sustainment of telehealth [20], and lack of provider acceptance is noted as the greatest barrier to widespread implementation of telehealth to date [21]. This suggests it may be fruitful to explore how significant circumstances that necessitate implementation impact attitudes toward implementation and innovation adoption.

In order to support rapid implementation, collaboration between researchers, funders, health systems workers, practitioners, and community partners toward a common cause is necessary [5]. Implementation efforts are most successful when propelled by the alignment of support across system and organizational contexts [22]. Historically, telehealth has been difficult to implement, scale-up, and sustain [3, 23] often due to lack of funding and/or policy support at the system-level [24, 25]. In the context of COVID-19, restrictions around the privacy of patient health information (i.e., HIPAA) and Medicaid billing requirements were adjusted, which ultimately aided the rapid transition to telehealth [26–29]. The sustained use of telehealth will likely be a function of continued billing capabilities and policies to facilitate sustainment.

To maximize the success of rapid implementation, the needs of a range of stakeholders, focusing on time-pressured, clinically relevant questions, must be considered [5]. Collecting and providing timely information of value to stakeholders (i.e., practitioners, decision-makers, and policy makers) can guide specific actions to support rapid implementation. In this study, results suggested that although providers perceived both therapeutic relationships and clients’ willingness to schedule telehealth sessions as somewhat better than in-person services, some providers reported difficulty maintaining engagement and using EBPs via telehealth. Providers also reported experiencing stress related to billing, documentation, and productivity demands while adapting to work changes and maintaining work-life balance. To sustain rapid implementation of telehealth services, it will be essential for CMHC leaders to consider this information and identify specific actions and supports that are needed such that providers can effectively engage with clients and deliver EBPs via telehealth [30, 31].

This study lends important insight into provider experiences with telehealth implementation and EBP delivery in the context of the COVID-19 outbreak. There are, however, several limitations. Due to the novel nature of this outbreak and the related work changes, some measures were created or adapted for this study and do not yet have published psychometrics, making cross-study comparison challenging. Also, this study did not include client- or treatment-level information, such as client perspectives on telehealth, nor did it include the perspectives of other relevant stakeholders, such as CMHC or system-level leadership. Additionally, though not directly assessed in this study, it is very likely that pandemic-related stressors and the associated impacts on cognitive functioning could have impacted the quality of the rapid implementation to telehealth. Finally, it is unclear how expeditious implementation will impact the sustainability of telehealth; moving too quickly through implementation without adequate planning may result in the omission of key implementation activities, and ultimately non-sustainment [32]. Future research should focus on the extent to which the rapid implementation of telehealth in the context of COVID-19 results in sustainment.

## Conclusion

In the context of the COVID-19 outbreak, CMHC providers continued to provide services and deliver EBPs through telehealth. Persistence was needed to connect and engage with clients, and creativity was crucial for continued EBP delivery. Though rapid telehealth implementation was supported by the relaxing of national policies, it remains unclear whether telehealth will be sustained moving forward, and, if so, what support providers need to do so effectively.

## Supplementary Information

**Additional file 1.** STROBE Statement—checklist of items that should be included in reports of observational studies.

## Data Availability

Not applicable

## References

[1] Wind TR, Rijkeboer M, Andersson G, Riper H. The COVID-19 pandemic: the ‘black swan’ for mental health care and a turning point for e-health. Internet Interv. 2020;20. 10.1016/j.invent.2020.100317. 10.1016/j.invent.2020.100317 PMC7104190 32289019

[2] Vis C, Mol M, Kleiboer A, Bührmann L, Finch T, Smit J (2018). Improving implementation of eMental health for mood disorders in routine practice: systematic review of barriers and facilitating factors. JMIR Ment Health..

[3] Greenhalgh T, Wherton J, Papoutsi C, Lynch J, Hughes G, Hinder S (2017). Beyond adoption: a new framework for theorizing and evaluating nonadoption, abandonment, and challenges to the scale-up, spread, and sustainability of health and care technologies. J Med Internet Res..

[4] Tuerk PW, Keller SM, Acierno R (2019). Treatment for anxiety and depression via clinical videoconferencing: evidence base and barriers to expanded access in practice. Focus..

[5] Smith J, Rapport F, O’Brien TA, Smith S, Tyrrell VJ, Mould EVA, Long JC, Gul H, Cullis J, Braithwaite J (2020). The rise of rapid implementation: a worked example of solving an existing problem with a new method by combining concept analysis with a systematic integrative review. BMC Health Serv Res..

[6] Balas EA, Boren SA, Bemmel J, McCray AT (2000). Managing clinical knowledge for health care improvement. Yearbook of medical informatics 2000: patient-centered systems.

[7] Fixsen DL, Blasé KA, Timbers GD, Wolf MM, Bernfeld GA, Farrington DP, Leschied AW (2007). In search of program implementation: 792 replications of the teaching-family model. Offender rehabilitation in practice: implementation and evaluating effective programs.

[8] Aarons GA, Ehrhart MG, Moullin JC, Torres EM, Green AE. Testing the leadership and organizational change for implementation (LOCI) intervention in substance abuse treatment: a cluster randomized trial study protocol. Implement Sci. 2017;12. 10.1186/s13012-017-0562-3. 10.1186/s13012-017-0562-3 PMC5335741 28253900

[9] Aarons GA. Statewide system and organizational strategy for evidence-based practice implementation and sustainment in adolescent substance use disorder treatment (R01DA049891): National Institute on Drug Abuse; 2020.

[10] Wu P, Fang Y, Guan Z, Fan B, Kong J, Yao Z, Liu X, Fuller CJ, Susser E, Lu J, Hoven CW. The psychological impact of the SARS epidemic on hospital employees in China: exposure, risk perception, and altruistic acceptance of risk. Can J Psychiatry. 2009;54:302–11. 10.1177/070674370905400504 PMC3780353 19497162

[11] LeNoble C, Horan K, Shoss M, Kwesell A. Effects of institutional responses to the COVID-19 pandemic on undergraduate faculty and students across STEM disciplines. In C. LeNoble (chair). Making I/O research RAPID in times of crisis: Insights into quick-response NSF funding. Symposium accepted to the 2021 annual SIOP conference. 2021.

[12] Coltman T, Devinney TM, Midgley DF, Venaik S (2008). Formative versus reflective measurement models: two applications of formative measurement. J Bus Res.

[13] Kristensen TS, Borritz M, Villadsen E, Christensen KB (2005). The Copenhagen burnout inventory: a new tool for the assessment of burnout. Work Stress..

[14] Eisenberger R, Stinglhamber F, Vandenberghe C, Sucharski IL, Rhoades L (2002). Perceived supervisor support: contributions to perceived organizational support and employee retention. J Appl Psychol..

[15] Lau AS, Brookman-Frazee L (2016). The 4KEEPS study: identifying predictors of sustainment of multiple practices fiscally mandated in children's mental health services. Implement Sci..

[16] Kim JJ, Brookman-Frazee L, Gellatly R, Stadnick N, Barnett ML, Lau AS (2018). Predictors of burnout among community therapists in the sustainment phase of a system-driven implementation of multiple evidence-based practices in children’s mental health. Prof Psychol Res Pr..

[17] Jex SM, Bliese PD (1999). Efficacy beliefs as a moderator of the impact of work-related stressors: a multilevel study. J Appl Psychol..

[18] Mace S, Boccanelli A, Dormond M. The use of telehealth within behavioral health settings. Utilization, opportunities, and challenges. Ann Arbor: University of Michigan School of Public Health; 2018.

[19] Crabtree BF, Miller WL, Crabtree BF, Miller WL (1999). Using codes and code manuals: a template organizing style of interpretation. Doing qualitative research 2nd edition.

[20] Wade VA, Eliott JA, Hiller JE (2014). Clinician acceptance is the key factor for sustainable telehealth services. Qual Health Res..

[21] Topooco N, Riper H, Araya R, Berking M, Brunn M, Chevreul K (2017). Attitudes towards digital treatment for depression: a European stakeholder survey. Internet Interv.

[22] Aarons GA, Ehrhart MG, Farahnak LR, Sklar M (2014). Aligning leadership across systems and organizations to develop a strategic climate for evidence-based practice implementation. Annual Review of Public Health.

[23] van Limburg M, van Gemert-Pijnen JE, Nijland N, Ossebaard HC, Hendrix RM, Seydel ER (2011). Why business modeling is crucial in the development of eHealth technologies. J Med Internet Res..

[24] Benavides-Vaello S, Strode A, Sheeran BC (2013). Using technology in the delivery of mental health and substance abuse treatment in rural communities: a review. J Behav Health Serv Res..

[25] Ross J, Stevenson F, Lau R, Murray E (2016). Factors that influence the implementation of e-health: a systematic review of systematic reviews (an update). Implement Sci..

[26] Office for Civil Rights. Bulletin: Civil rights, HIPAA, and the coronavirus disease 2019 (COVID-19). U.S. Department of Health and Human Services. 2020. https://www.hhs.gov/sites/default/files/ocr-bulletin-3-28-20.pdf. Accessed 1 Sept 2020.

[27] Office for Civil Rights. FAQs on Telehealth and HIPAA during the COVID-19 nationwide public health emergency. U. S. Department of Health and Human Services. 2020. https://www.hhs.gov/sites/default/files/telehealth-faqs-508.pdf. Accessed 1 Sept 2020.

[28] Office for Civil Rights. Notification of enforcement discretion for telehealth remote communications during the COVID-19 nationwide public health emergency. U.S. Department of Health and Human Services. 2020. https://www.hhs.gov/hipaa/for-professionals/special-topics/emergency-preparedness/notification-enforcement-discretion-telehealth/index.html. Accessed 1 Sept 2020.

[29] Center for Medicare & Medicaid Services. State Medicaid & CHIP telehealth toolkit: policy considerations for states expanding use of telehealth: COVID-19 version. U.S. Department of Health and Human Services. 2020. https://www.medicaid.gov/medicaid/benefits/downloads/medicaid-chip-telehealth-toolkit.pdf. Accessed 1 Sept 2020.

[30] Gros DF, Morland LA, Greene CJ, Acierno R, Strachan M, Egede LE (2013). Delivery of evidence-based psychotherapy via video telehealth. J Psychopathol Behav Assess..

[31] Gagnon MP, Duplantie J, Fortin JP, Landry R (2006). Implementing telehealth to support medical practice in rural/remote regions: what are the conditions for success?. Implement Sci..

[32] Saldana L, Chamberlain P, Wang W, Brown CH (2012). Predicting program start-up using the stages of implementation measure. Adm Policy Mental Health..

